# Cancer survivors’ interests and preferences for muscle-strengthening activity interventions

**DOI:** 10.1007/s00520-026-11312-7

**Published:** 2026-10-09

**Authors:** Julia Pincever, Julia Frey, Payton Solk, Jean M. Reading, Shirlene Wang, Hannah Freeman, Melanie Wolter, Brendan Hickey, Lauren Wang, Fiona Webb, Faye M. Walker, Ria Desai, Siobhan M. Phillips

**Affiliations:** 1 Department of Preventive Medicine, Northwestern University Feinberg School of Medicine, 680 N Lake Shore Drive, Suite 1400, Chicago, IL 60611 USA; 2 Department of Family and Community Medicine, University of Illinois at Chicago College of Medicine, Chicago, IL USA

**Keywords:** Exercise, Cancer survivor, Muscle strengthening, Resistance training, Behavioral medicine

## Abstract

**Purpose:**

Muscle-strengthening activities (MSA) are associated with improved health outcomes in cancer survivors. Yet, less than 20% of cancer survivors report meeting MSA guidelines (i.e., full body MSA ≥ 2 times/week). Technology-supported MSA interventions may enhance adherence to activity guidelines. This study examined cancer survivors’ interests and preferences for MSA interventions.

**Methods:**

Post-treatment cancer survivors [*N* = 556; Mage = 56.5(SD = 14.7); 53.6% female; 49.3% met MSA guidelines] within 5 years of diagnosis completed questionnaires to assess MSA participation and interests, and preferences for MSA interventions. A subset (*n* = 30; 50% met MSA guidelines) was randomly selected to complete a semi-structured interview. Quantitative data were analyzed using descriptive statistics. Interviews were recorded, transcribed, and analyzed using an iterative thematic content analysis approach and consensus review.

**Results:**

Participants indicated they would be interested in participating in a MSA (67.6%) intervention tailored to cancer survivors, but preferences varied across potential features. Five themes captured participant preferences from interview data: (1) Participants believe MSA interventions could be useful for improving health and physical functioning; (2) Many multi-level barriers and facilitators to MSA participation exist; (3) Prior experience with MSA guided current intervention preferences; (4) Desire for support and accountability; (5) Need for convenient, flexible interventions with varied preferences for delivery modality, structure, and personalization.

**Conclusions:**

Cancer survivors are interested in MSA interventions, but preferences for intervention features vary. Results from this study can help inform future MSA intervention development to create acceptable, feasible, and effective programs.

**Supplementary Information:**

The online version contains supplementary material available at https://doi.org/10.1007/s00520-026-11312-7.

## Introduction

There are approximately 18.6 million cancer survivors (survivors) in the U.S [1]. This number is expected to surpass 22 million over the next decade [1]. Cancer and its treatment are associated with negative side effects (e.g., fatigue, depression, anxiety, decreased physical functioning), increased risk for chronic conditions, and compromised quality of life (QOL) [2–8]. Participation in muscle-strengthening activities (MSA) has been associated with improved anxiety and fatigue, chronic pain, sleep behaviors, physical function, self-esteem, QOL, strength capacity and decreased falls risk among survivors [9–13]. Emerging data indicate increased MSA participation may be associated with reduced cancer mortality [14–16] recurrence [15, 17]. As a result, The American College of Sports Medicine recommends survivors engage in full body MSA at least two days per week [18]. However, only 19.2% of survivors report meeting strength-based guidelines [19] and engage in less MSA than those without a cancer history (30.8%, women; 39.5%, men) [20]. This is concerning because lean mass, muscular strength, and endurance decline during cancer treatment while fat mass and frailty risk increases [21, 22]. Low muscle mass is also associated with worse clinical outcomes post-treatment [23]. Thus, it is critical to better understand survivors’ interests and preferences for MSA interventions to facilitate effective intervention development and increase MSA.

Although several studies have explored survivors’ interests and preferences for physical activity (PA) interventions, few have focused on MSA interventions [24–27]. Understanding MSA behavior alongside moderate and vigorous aerobic physical activity (MVPA) is important to comprehensively understand cancer surviviors’ adherence to physical activity guidelines and more accurately characterize the benefits of different types of physical activity. While many barriers to MSA and MVPA are shared (i.e., perceived lack of time, low self-efficacy, cost), there are many unique barriers to MSA [28]. Compared to aerobic activities like running or walking, MSA is often perceived as more complex, requiring specialized equipment, knowledge, or technique instruction which may limit uptake among survivors with comorbid conditions or may heighten concerns about safety and injury [28, 29]. Thus, focusing specifically on MSA intervention interests and preferences is warranted to enhance intervention feasibility and acceptability and to maximize benefit. The purpose of the present study is to better understand survivors’ interests and preferences for MSA intervention components and features to inform future MSA interventions.

## Methods

### Participants and procedures

This study was a cross-sectional mixed methods observational study. Participants were recruited from Northwestern electronic medical records via the Northwestern Medicine Electronic Data Warehouse in proportions similar to the U.S. cancer survivor population for cancer type and age [1]. Inclusion criteria included self-reported (1) age ≥ 18 years old; (2) non-metastatic cancer diagnosis; (3) ≤ 5 years since cancer diagnosis; (4) ≥ 3 months post–primary cancer treatment completion (surgery, radiation, and/or chemotherapy); (5) internet access; (6) ability to read/write in English; and (7) not currently pregnant. Participants completed screening online. Eligible participants were redirected to complete informed consent followed by questionnaires and sent up to 3 reminders to complete each.

Following questionnaire completion, a subsample (*n* = 30) who indicated interest in participating in an interview on their informed consent were randomly selected using a pre-populated computer algorithm stratified by meeting MSA recommendations (50% engaging in ≥ 2 days MSA/week) to ensure the sample had a range of activity levels and familiarity with physical activity, age (≥ 60, < 60), and gender to capture preference diversity and achieve saturation. Participants were emailed and called up to 3 times to schedule an interview.

### Measures

#### Demographics

Participants self-reported demographics (age (years), gender, marital status, income, race, ethnicity), health (height, weight overall health rating, comorbid conditions), and cancer history (diagnosis date and age, disease stage, treatment and duration).

#### Muscle-Strengthening Exercise Questionnaire (MSEQ-short form)[30]

The 6-item MSEQ assessed MSA participation (yes/no). For those indicating MSA participation, usual MSA frequency, session duration, intensity, participation (yes/no) in four MSA types (weight machines, bodyweight exercises, resistance exercises and holistic exercises), and muscle groups targeted (yes/no) were assessed. The MSEQ has high test–retest reliability (*ρ* range = 0.76–0.91) and moderate to high concurrent validity (*ρ* range = 0.30–0.77) for frequency, duration, and intensity. We used the frequency item to determine (yes/no) whether MSA guidelines (≥ 2 days/week) were met [18].

#### Godin Leisure-Time Exercise Questionnaire (GLTEQ)[31]

A modified version of the GLTEQ, a valid and reliable assessment tool for MVPA in survivors that is highly correlated with accelerometer-assessed MVPA, was used to assess current, weekly strenuous, moderate and light intensity aerobic activity frequency for more than 15 min and the average minutes each session [32]. The frequency for moderate and strenuous activities was multiplied by session time and summed to estimate average weekly MVPA minutes and classify whether participants met (yes/no) MVPA guidelines (i.e., 150 min/week) [18].

#### MSA intervention beliefs, interests and preferences

##### Questionnaire

Participants completed modified questions from prior, similar studies [33–35]. The questionnaire defined MSA and then assessed whether they believed survivors should do MSA, interest in participating in an MSA program for survivors, physical ability to do MSA and preferences for frequency, intensity, duration, type, format (location, delivery modality), time point in cancer continuum, supervision level, counseling, and self-monitoring for in-person and technology-supported MSA interventions. Participants rated how helpful they believed 18 potential MSA intervention components would be on a scale from 1 (not helpful) to 5 (very helpful).

##### Interviews

Staff members trained in PA and cancer survivorship conducted interviews via Zoom. Staff followed a semi-structured interview guide developed by the research team. The questions assessed participants’ PA history, barriers and facilitators, and interests and preferences for MSA interventions (see Supplementary Table 1). Interviews lasted 20–45 min and were recorded, transcribed, and deidentified before qualitative analysis.

### Data analyses

Demographic, PA, and intervention preferences data were analyzed using descriptive statistics including means and standard deviations (continuous variables) and frequencies (categorical variables). Linear regression was used to determine whether participant characteristics including: gender (female v. male), race (White v. non-White); ethnicity (Hispanic/LatinX v. Non-Hispanic/LatinX); income (< 50 k v. > 50 k); age (continuous); education (continuous); BMI (overweight/obese v. normal weight); treatments received (i.e. chemotherapy, radiation, immunotherapy, surgery; yes v. no), health status (continuous), meeting MSA guidelines (yes v. no) or meeting MVPA guidelines (yes v. no) influenced preferences. All analyses were conducted in SPSS 30.0 (IBM Corp, Armonk, NY).

For interview data, key themes were identified via thematic text analysis using an iterative inductive approach [36, 37]. Two research team members (JP, MW) identified key concepts from interview transcripts, compared content codes and iteratively developed a coding scheme (code names and meanings) to describe and summarize relevant content. The coding scheme was reviewed by the larger research team to achieve consensus. Two study team members (among JP, MW, BH, GA) independently read transcripts and assigned codes to relevant content. Coding discrepancies were resolved via discussion [38]. The study team organized codes into broader categories based on conceptual similarities and a priori research questions. Consensus was reached via discussion. Pairs of authors (PS, RD, JP) independently reviewed the narrative content within each specific thematic category, summarized findings and identified illustrative quotes. Each pair reviewed and resolved any inconsistencies through iterative consensus [39]. Dedoose 9.0.17 (SocioCultural Research Consultants, Los Angeles, CA) was used to organize and code qualitative data within thematic categories.

## Results

### Participants

A total of 759 participants consented to the study; 673 started the survey and 556 participants had complete data on the MSA Intervention Beliefs, Interests and Preferences questionnaire and were included in the present analyses. 553 participants completed the MSEQ- short form. A total of 450 participants finished the full survey of which 30 were randomized and completed the interview. On average, survey participants were 56.5 years old (SD = 14.7), had an average BMI of 28.4 (SD = 0.3), and were 3.8 years since diagnosis. 53.6% were female. The majority were White (76.4%), Non-Hispanic (86.3%), had ≥ college degree (79.1%) and were employed full-time or part-time (58.1%). The most common cancer diagnoses were breast (21.4%), prostate (19.2%), lung (5.9%), colon (5.6%), and endometrial (5.0%). Cancer treatments received included surgery (84.7%), chemotherapy (40.5%), radiation (43.3%), immunotherapy (10.4%), and stem cell transplant (3.6%); 15.5% reported they were currently receiving hormone therapy. Most participants rated their overall health status as excellent/very good (44.8%) or good (40.5%) and 99.1% owned a smartphone. Participants engaged in MSA an average of 1.7 (SD = 1.9) days per week for 20.2 (SD = 30.5) min per day and an average of 174.1 (SD = 184.5) min/week of MVPA. About one-third (33.6%) met both MSA (i.e., 2 days per week) and MVPA (i.e., 150 min/week) guidelines, 15.9% met MVPA guidelines only, 15.7% met MSA only, and 34.7% did not meet either guideline. About half of the participants (45.3%) did not participate in any MSA, 18.3% did not participate in any MVPA or MSA, and 2.5% participated in 1–149 min of MVPA and 1 day/week of MSA. Participants who completed interviews had similar demographics to the full sample (See Table 1).

**Table 1:** Demographics of cancer survivors

Variable	All participants (*n* = 556)	Interview participants (*n* = 30)
Age (***M***, SD)	56.5 (14.7)	59.2 (13.2)
BMI (***M***, SD)	28.4 (0.3)	28.6 (0.9)
Gender (***n***, %)		
Female	298 (53.6%)	14 (46.6%)
Male	256 (46.0%)	15 (50.0%)
Other	2 (0.4%)	1 (3.3%)
Race (***n***, %)		
White	425 (76.4%)	22 (73.3%)
Black or African American	76 (13.7%)	4 (13.3%)
Asian	53 (9.5%)	3 (10.0%)
Other or Unknown	20 (3.6%)	1 (3.3%)
Hispanic (***n***, %)	76 (13.7%)	2 (6.6%)
Employment status (***n***, %)		
Working Full Time	275 (49.5%)	15 (50.0%)
Working Part-Time	48 (8.6%)	2 (6.7%)
Retired	187 (33.6%)	9 (30.0%)
Other	45 (8.3%)	4 (13.3%)
Education (***n***, %)		
< College Degree	116 (20.9%)	9 (30.0%)
College Degree	216 (38.8%)	8 (26.7%)
Graduate Degree	224 (40.3%)	13 (43.3%)
Household income (***n***, %)		
< $50,000	83 (14.9%)	10 (33.3%)
> $50,000	452 (81.3%)	20 (66.7)
Prefer not to say	21 (3.8%)	0 (0.0%)
Own smartphone (***n***, %)	551 (99.1%)	30 (100.0%)
Time since diagnosis (years) (***M***, SD)	3.8 (3.6)	3.3 (2.8)
Type of cancer (***n***, %)		
Breast	119 (21.4%)	4 (13.3%)
Prostate	107 (19.2%)	6 (20.0%)
Lung	33 (5.9%)	3 (10.0%)
Colon	31 (5.6%)	6 (20.0%)
Endometrial	28 (5.0%)	0 (0.0%)
Non-Hodgkins Lymphoma	21 (3.8%)	4 (13.3%)
Urinary	43 (7.8%)	2 (6.6%)
Bone	4 (0.7%)	2 (6.6%)
Oral	2 (0.4%)	1 (3.3%)
Other	79 (14.2%)	2 (6.7%)
Cancer treatment received (***n***, %)		
Surgery	471 (84.7%)	27 (90.0%)
Chemotherapy	225 (40.5%)	12 (40.0%)
Radiation therapy	241 (43.3%)	12 (40.0%)
Immunotherapy	58 (10.4%)	3 (10.0%)
Hormone therapy	67 (12.1%)	4 (13.3%)
Targeted therapy (stem cell)	20 (3.6%)	1 (3.3%)
Other	52 (9.4%)	1 (3.3%)
Current treatment (***n***, %)		
Hormone therapy	86 (15.5%)	5 (16.7%)
Overall health status (***n***, %)		
Excellent/very good	249 (44.8%)	17 (56.7%)
Good	225 (40.5%)	11 (36.7%)
Fair/poor	82 (14.7%)	2 (6.7%)
Muscle strengthening activity (***n*** = 553)		
Average number of sessions per week (*M,* SD)	1.7 (1.9)	3.3 (1.1)
Average minutes day (*M,* SD)	20.2 (30.5)	15 (33.6)
Moderate to vigorous physical activity		
Average Minutes Per week (*M*, SD)	174.1 (184.5)	178.5 (143.1)
Physical activity guidelines (***n*** = 553)		
Meet MSA Guidelines only (≥ 2 times/week) (*n*, %)	87 (15.7%)	5 (16.7%)
Meet MVPA Guidelines only (≥ 150 min/week) (*n*, %)	88 (15.9%)	7 (23.3%)
Meet both MSA and MVPA Guidelines (≥ 150 min MVPA/week, ≥ 2 times/week) (*n*, %)	186 (33.6%)	10 (33.3%)
Do Not Meet MSA or MVPA Guidelines (≥ 150 min/week MVPA, ≥ 2 times/week MSA) (*n*, %)	192 (34.7%)	8 (26.7%)

### General MSA intervention beliefs, interests and preferences

Questionnaire data are presented in Table 2. Almost all (98.6%) participants indicated yes/maybe survivors should do MSA; only 20% indicated their oncologist recommended MSA. Most indicated they were (67.6%) or were maybe (25.2%) interested in an MSA program for survivors and believed they were (78.2%) physically able to participate in an MSA intervention. Participants indicated it would be somewhat/very helpful to do an MSA program at all time points on the cancer care continuum (range 70.4% to 92.6%) with the highest endorsement (92.6%) for both 6-months and 6-months to 1-year post-treatment. The top three MSA outcomes participants were interested in achieving were increased muscle tone (80.4%), strength (80.2%), and energy (77.9%). Most participants were interested (61.9%) in a structured (specific days/times) program. They were willing to spend maximum 3–4 days/week doing MSA (56.7%) for 20–39 min per session (49.7%) and preferred one longer daily session (68.7%) rather than multiple shorter sessions. Participants favored medium-intensity MSA (70.3%) with a mix of light weights with high repetitions and heavy weights with lower repetitions (60.3%). Most participants (41.9%) preferred an MSA program offering variety through a mixture of split training (i.e. separate days for separate body parts) and full body days mixed with cardio. They reported interest in a range of MSA types; most popular options were free weights (65.3%), mix of free weights and resistance bands (51.8%), yoga (49.3%), resistance bands (46.9%), and balance exercises (45.7%).

**Table 2:** General MSA intervention beliefs and preferences results

Item	Rating [*n* (%)]
Believe cancer survivors should engage in regular MSA	
Yes	473 (85.1%)
Maybe	75 (13.5%)
No	8 (1.4%)
Oncologist ever recommended MSA	
Yes	111 (20.0%)
Not Sure	86 (15.5%)
No	359 (64.6%)
Interest in an MSA program specifically for cancer survivors	
Yes	376 (67.6%)
Maybe	140 (25.2%)
No	40 (7.2%)
Physically able to participate in an MSA intervention	
Yes	435 (78.2%)
Maybe	106 (19.1%)
No	15 (2.7%)
Timepoint to engage in MSA (% somewhat helpful/very helpful)	
Before treatment	431 (77.6%)
During treatment	391 (70.4%)
Immediately post-treatment	433 (77.9%)
6-months post-treatment	515 (92.6%)
6-months to 1-year post-treatment	515 (92.6%)
More than 1-year post-treatment	494 (88.8%)
Outcomes would like to achieve from MSA program (check all that apply)	
Increased muscle tone	447 (80.4%)
Increased strength	446 (80.2%)
Increased energy	433 (77.9%)
Mental well-being benefits	391 (70.3%)
Increased balance	383 (68.9%)
Weight loss	341 (61.3%)
Fit in clothes better	280 (50.4%)
Structure of MSA training program	
Structured (specific days/times)	344 (61.9%)
Spontaneous/flexible	151 (27.2%)
No preference	61 (11.0%)
Maximum number of days willing to participate in MSA	
0–2 days per week	85 (15.3%)
3–4 days per week	315 (56.7%)
5–6 days per week	125 (22.4%)
7 days per week	31 (5.6%)
Length of each MSA sessions	
1–19 min	41 (7.4%)
20–39 min	276 (49.7%)
40–59 min	169 (30.4%)
> 60 min	70 (12.6%)
MSA sessions per day	
One long workout a day	382 (68.7%)
Shorter sessions a couple of times a day	174 (31.3%)
Intensity of MSA session (check all that apply)	
Light	123 (22.1%)
Medium	391 (70.3%)
High	124 (22.3%)
Weight and repetition (reps)	
Mix of light weights + high reps/heavy weights + lower reps	335 (60.3%)
Light weights + high reps	178 (32.0%)
Heavy weights + low reps	43 (7.7%)
Muscle group focus structure	
Mixture of separate days for separate body parts and full body days mixed with cardio	233 (41.9%)
Separate days for separate body parts	134 (24.1%)
Mixture of separate days/separate body parts and full body	124 (23.3%)
Full body only	65 (11.7%)
Type of MSA interested in (Check all that apply)	
Free Weights	363 (65.3%)
Weights and resistance bands	288 (51.8%)
Yoga	274 (49.3%)
Resistance bands	261 (46.9%)
Weights	254 (45.7%)
Balance exercises	254 (45.7%)
Bodyweight	214 (38.5%)
Pilates	179 (32.2%)
Rowing machine	150 (27.0%)
Boxing/kickboxing	121 (21.8%)
Dancing	107 (19.2%)
Barre	70 (12.6%)
No preference	35 (6.3%)
Preference on individual or group MSA sessions	
Combination of individual and group sessions	214 (38.5%)
Alone	194 (34.9%)
With others in a group (in-person or online)	64 (11.5%)
Neither	84 (15.1%)
MSA session supervision preferences	
Exercise specialist in person	187 (33.6%)
Unsupervised but guided by guidebook, app or videos	143 (25.7%)
Supervised for initial session then unsupervised	110 (19.8%)
Exercise specialist virtually	64 (11.5%)
Completely unsupervised	49 (8.8%)
No preference	46 (8.3%)
Interest in 1-on-1 MSA instruction/coaching	
Yes	337 (60.6%)
No	58 (10.4%)
Maybe	161 (29.0%)
Preferred MSA instruction location	
In home via smartphone app, Zoom or other web-based video service	84 (34.9%)
At a fitness club	161 (29.0%)
At a cancer center or hospital	84 (15.1%)
At a community center or YMCA	37 (6.7%)
Streaming through TV, Roku or Amazon Stick	32 (5.8%)
Other type of instruction	24 (4.3%)
Not interested in instruction	24 (4.3%)
Preferences for who should deliver MSA behavior change counseling (check all that apply)	
Exercise Specialist within Cancer Center	252 (45.3%)
Physical Therapist	161 (29.0%)
Personal Trainer	142 (25.5%)
No Preference	127 (22.8%)
Oncologist	113 (20.3%)
Exercise Specialist at a Community Center or Health Club	107 (19.2%)
Primary Care Physician	94 (16.9%)
Cancer Patient or Survivor	50 (9.0%)
Nurse	34 (6.1%)
Other	9 (1.6%)
Preferred modality for monitoring MSA progress	
Fitbit/Apple Watch	251 (45.1%)
Routine strength tests	125 (22.5%)
Functional performance testing	92 (16.5%)
Handwritten fitness log	88 (15.8%)
Feedback interested in receiving during MSA program (check all that apply)	
Progress	396 (71.2%)
Form	321 (57.7%)
Exercise modifications	293 (52.7%)
Heart rate during workouts	187 (33.6%)
Difficulty of workout	194 (34.9%)
Would use Tonal, Tempo, or Peloton if available at cancer center for free	
Yes	253 (45.5%)
Maybe	215 (38.7%)
No	88 (15.8%)
Would use gym with weights if available for free at cancer center	
Yes	231 (41.5%)
Maybe	191 (34.4%)
No	134 (24.1%)
MSA equipment currently have at home (check all that apply)	
Free weights	394 (70.9%)
Yoga equipment (i.e., mat, blocks, strap)	266 (47.8%)
Loop resistance bands (i.e., wrap around your ankles, calves, arms)	261 (46.9%)
Standard resistance bands (i.e., long band with handle)	192 (34.5%)
Core resistance equipment (i.e., bosu ball, exercise ball)	171 (30.8%)
Weight machines (i.e., squat rack)	67 (12.1%)
Amount willing to pay for MSA program per month	
$0–$25	325 (58.5%)
$26–$50	133 (23.9%)
$51–$100	80 (14.4%)
$100–$200 +	18 (3.2%)
Preference for in-person v. remotely delivered MSA Program	
Combination of in-person and remotely-delivered/home-based	265 (47.7%)
In-person	152 (27.3%)
Remotely delivered/home-based	129 (23.2%)
Neither	10 (1.6%)
Preference for remotely delivered MSA program modality (check all that apply)	
On demand	266 (47.8%)
Combination of live video and on demand	239 (43.0%)
Live one-on-one via videoconference	152 (27.3%)
Smartphone application	189 (34.0%)
Live group training via videoconference	72 (12.9%)
Would you use a commercially available fitness app if given free access?	
Yes	355 (63.8%)
Maybe	173 (31.1%)
No	28 (5.0%)
Would you use a free app designed to increase MSA for Cancer Survivors?	
Yes	389 (70.0%)
Maybe	145 (26.1%)
No	22 (4.0%)
Do you use an MSA application already?	
Yes	199 (35.8%)
No	357 (64.2%)
Time willing to spend engaging with fitness app for MSA per day	
1–29 min	243 (43.7%)
30–49 min	207 (37.2%)
50 + minutes	106 (19.1%)
Interested in trying “smart” commercial MSA equipment (% Yes)	
Tonal	362 (65.1%)
Peloton	323 (58.1%)
Tempo	310 (55.8%)

Respondents preferred a combination of individual and group sessions (38.5%) or all individual sessions (34.9%). Preferences for MSA supervision varied. The most popular options were supervision by an exercise specialist in person (33.6%), unsupervised but guided by a guidebook, app or videos (25.7%) and initial session supervision and then unsupervised (19.8%). The majority were also interested in some form of 1-on-1 coaching/instruction (89.6%). They were most interested in receiving MSA coaching/instruction at home via web-based video service (34.9%) followed by a fitness club (29.0%) and a cancer center or hospital (15.1%). Participants preferred MSA behavior change counseling from an exercise specialist at the cancer center (45.3%), physical therapist (29.0%), or a personal trainer (25.5%). Participants were interested in receiving feedback on their MSA progress (71.2%) and a wearable device (e.g. Fitbit/Apple Watch) (45.1%) was the most favored modality. Participants were keen on receiving feedback on MSA form (57.7%), and modifications (52.7%).

Some indicated they would use a gym with weights (41.5%) or commercially available “smart” connected equipment (45.5%) if available for free at the cancer center. Most participants owned free weights (70.9%) and would be willing to spend between $0 and $50/month (82.4%) on an MSA program**.** Almost half (47.7%) preferred a combined in-person and technology-supported MSA intervention; the other half were split between wanting all in-person (27.3%) and all technology-supported (23.2%). Participants were most interested in an on-demand (47.8%) or live and on-demand combination (43%) format for a technology-supported MSA program. Most participants indicated yes/maybe they would use a commercially available fitness smartphone application (i.e., app) if given free access (94.9%) or a free app designed to increase MSA (96.1%) for survivors; 35.8% were currently using an MSA app. Most participants were willing to spend 1–29 min (43.7%) engaging with an MSA app. Participants were generally interested in trying “smart” connected equipment including Tonal (65.1%), Peloton (58.1%), and Tempo (55.8%).

### Preferences for specific MSA intervention components

Data are presented in Table 3. Components rated as most helpful included the following: in-person 1-on-1 (83.6%) and group (76.3%) MSA training sessions, activity tracker (71.6%) and website with MSA videos specifically designed for survivors (71.4%). Components rated as least helpful were social networking sites integration (20.3%), emails (36.7%), and telephone counseling sessions (38.4%).

**Table 3:** Interest in specific potential MSA program features

In person 1-on-1 MSA training sessions	Rating [*n* (%)]
Somewhat/very helpful	465 (83.6%)
Not sure	54 (9.7%)
Not/Not very helpful	37 (6.7%)
In-person group MSA training sessions	
Somewhat/very helpful	424 (76.3%)
Not sure	68 (12.2%)
Not/Not very helpful	64 (11.5%)
1-on-1 videoconference MSA training sessions	
Somewhat/very helpful	375 (67.4%)
Not sure	102 (18.3%)
Not/Not very helpful	79 (14.3%)
Group videoconference MSA training sessions	
Somewhat/very helpful	282 (50.7%)
Not sure	147 (26.4%)
Not/Not very helpful	127 (22.8%)
Website with MSA videos designed for cancer survivors	
Somewhat/very helpful	397 (71.4%)
Not sure	101 (18.2%)
Not/Not very helpful	56 (10.1%)
Smartphone app for MSA designed for cancer survivors	
Somewhat/very helpful	376 (67.6%)
Not sure	100 (18.0%)
Not/Not very helpful	80 (14.4%)
Smartphone app with MSA training videos designed for cancer survivors	
Somewhat/very helpful	376 (67.6%)
Not sure	107 (19.2%)
Not/Not very helpful	73 (13.1%)
Activity tracker	
Somewhat/very helpful	398 (71.6%)
Not sure	101 (18.2%)
Not/Not very helpful	57 (10.3%)
Access to “smart” MSA equipment	
Somewhat/very helpful	373 (67.0%)
Not sure	123 (22.1%)
Not/Not very helpful	60 (10.8%)
Telephone counseling sessions with an exercise specialist	
Somewhat/very helpful	213 (38.4%)
Not sure	154 (27.7%)
Not/Not very helpful	113 (20.3%)
Video conference counseling session with an exercise specialist	
Somewhat/very helpful	319 (57.3%)
Not sure	124 (22.3%)
Not/Not very helpful	113 (20.3%)
Personalized, encouraging text messages	
Somewhat/very helpful	232 (41.7%)
Not sure	134 (24.1%)
Not/Not very helpful	190 (34.2%)
Personalized, encouraging emails	
Somewhat/very helpful	204 (36.7%)
Not sure	138 (24.8%)
Not/Not very helpful	214 (38.5%)
Integrations with electronic health record (EHR)	
Somewhat/very helpful	306 (55.0%)
Not sure	158 (28.4%)
Not/Not very helpful	93 (16.6%)
MSA activity data shared with doctor	
Somewhat/very helpful	332 (59.7%)
Not sure	148 (26.6%)
Not/Not very helpful	76 (13.7%)
Exercise buddy from personal life to keep you accountable	
Somewhat/very helpful	288 (51.8%)
Not sure	119 (21.4%)
Not/Not very helpful	149 (26.8%)
Exercise buddy from MSA activity program to keep you accountable	
Somewhat/very helpful	293 (52.7%)
Not sure	120 (21.6%)
Not/Not very helpful	143 (25.8%)
Integration with social networking sites	
Somewhat/very helpful	113 (20.3%)
Not sure	157 (28.2%)
Not/Not very helpful	286 (51.5%)

### Preferences and participant characteristics

The relationship between preferences for specific MSA intervention features (Table 3) and demographic and disease characteristics is presented in Supplementary Table 2. Females rated 1-on-1 (*β* = 0.43; *p* = 0.01) and group “live” (*β* = 0.49; *p* < 0.001) videoconference MSA sessions, text messages (*β* = 0.41; *p* = 0.02), and emails (*β* = 0.39; *p* = 0.04) as more helpful than males. White participants perceived in-person individual (*β* = −0.32; *p* = 0.03) and group (*β* = −0.38; *p* = 0.01) MSA sessions, 1-on-1 videoconference MSA training sessions (*β* = −0.325, *p* = 0.01) and having a personal (*β* =−0.61; *p* < 0.001) and program (*β* = −0.66; *p* < 0.001) buddy as less helpful. Non-Hispanic/Latinx participants rated in-person group MSA sessions (*β* = 0–0.61; *p* = 0.001), activity trackers (*β* = −0.43; *p* = 0.02), EHR integration (*β* = −0.55; *p* = 0.01), and having a personal (*β* = −0.46; *p* = 0.04) and program (*β* =−0.53; *p* = 0.02) buddy as less helpful than Hispanic/Latinx participants. Older age was associated with lower perceived helpfulness of 1-on-1 MSA videoconference sessions (*β* = −0.01; *p* = 0.01), smartphone apps (*β* = −0.03; *p* < 0.001), MSA videos (*β* = −0.03, *p* < 0.001), activity trackers (*β* = −0.02; *p* < 0.001), “smart” MSA equipment (*β* = −0.03; *p* < 0.001), and a personal buddy (*β* = −0.02, *p* < 0.001). Higher education was associated with lower ratings of social networking integration (*β* = −0.18; *p* = 0.03) and emails (*β* = −0.27; *p* = 0.001). A household income of ≤ $100 k was associated with higher perceived helpfulness of social networking integration (*β* = 0.39; *p* < 0.001), personalized text messages (*β* = 0.36; *p* < 0.001) and emails (*β* = 0.35; *p* = 0.001). Obese or overweight individuals rated EHR integration (*β* = 0.80; *p* = 0.04) as more helpful than individuals with standard BMIs. Participants who received chemotherapy rated personal (*β* = −0.46; *p* = 0.01) or program (*β* = −0.43; *p* = 0.01) buddies less favorably than those who had not. In contrast, compared to those who had not received radiation, those with a history of radiation rated smart equipment (*β* = 0.29; *p* = 0.04) and program buddies (*β* = 0.35; *p* = 0.03) as more helpful. Participants who had surgery rated 1-on-1 in-person MSA sessions (*β* = 0.41; *p* = 0.04) and smart MSA equipment (*β* = 0.42; *p* = 0.01) as more helpful than those who had not. History of immunotherapy, health status, and meeting MSA or MVPA guidelines were not associated with specific feature ratings.

### Interview results

Our analysis identified five main themes that captured participants’ perspectives (see Table 4).

**Table 4:** Relevant quotes for each theme

Theme	Quote
Cancer survivors believe MSA interventions could improve health and physical functioning	“Because, as I said is, I*’*ve aged, you know, start losing your muscle mass. I think any form of exercise… I*’*ve seen for myself what it*’*s done for me over the past 5 years. It*’*s increased my stamina. It*’*s made me more social. Less depressed. I*’*m looking forward to things.” –female, 71, colon cancer, does not meet MSA guidelines
	“So that*’*s where you are, after you finish your treatment. You have to move backwards. So you need to get your muscles stand back.”- female 50, non-hodgkin lymphoma, meets MSA guidelines
Many multi-level barriers and facilitators exist for MSA participation	“Lack of equipment to do the exercise. The not having access to gyms because of the cost, because you*’*re spending just enormous amounts of your money that are going towards the hospital bills, doctors, treatments, prescriptions.” – male, 57, melanoma, does not meet MSA guidelines
Prior experience and familiarity with MSA guided current preferences for MSA interventions	“I probably would try the weights more so than the resistance bands. I might have seen those in action before and anything else along with the weights.” – male, 58, prostate cancer, does not meet MSA guidelines
Desire for support and accountability	“Yeah. I think community makes a big difference in those such things having people that you know, have the same interest in and same sort of challenges, definitely helpful with that sort of stuff.” – male, 31, leukemia/blood cancer, meets MSA guidelines
	“I need that support. I think a lot of people do and the accountability.” – female, 65, breast cancer, meets MSA guidelines
Need for flexible, convenient interventions with varied delivery, structure, and personalization	“I tried too early because I got ambitious, and I wanted to do it, and I went to the gym in December, and I couldn*’*t get up for 3 days. I was so sore. So I pushed myself up too early. That was not a good idea.” –female 50, non-hodgkin lymphoma, meets MSA guidelines
	“I would say that probably muscle atrophy, higher levels of pain, being tired for medications, trying to heal and that when you actively begin exercises it exacerbates what kind of surgeries and medications you*’*re taking to the point where it hurts too much.” –male, 57, melanoma, does not meet MSA guidelines
	“Not so much that, but I think I would attempt, if such data exist, to demonstrate factually the benefits of exercising in cancer treatment. Because if people you know, sometimes a generalization doesn*’*t motivate you, but when you look at statistics that demonstrate the benefit of it, I think you would get more people to become involved in it.” – male, 76 years old, lung cancer, meets MSA guidelines
	“The other thing for me is, I tend to try to work out where I was before and I need to find some sort of building process workout that isn*’*t like, hey, jump into a 30 min advanced workout right now, because I*’*m not going to be able to finish it.”- male, 49, bladder cancer, meets MSA guidelines

#### Theme 1: Survivors believe MSA interventions could improve health and physical functioning

Overall, participants believed MSA interventions would be useful for improving health and physical functioning. Participants thought starting MSA after completing primary treatment would be optimal to rebuild strength and improve physical functioning, mobility, and longevity, although some mentioned an intervention throughout cancer and its treatment would be beneficial. Participants noted cancer and its treatment had many negative side effects and believed MSA could help survivors return to pre-diagnosis physical functioning levels and prevent further declines.

#### Theme 2: Many multi-level barriers and facilitators exist for MSA participation

While many participants noted motivation as a facilitator to MSA participation, many identified it as a barrier. Participants reported limited intrinsic motivation to engage in MSA independently, but they thought motivation would increase if accountability and social support were provided. Knowledge was also cited as a barrier and facilitator to engaging in MSA. Many participants indicated they were unfamiliar with MSA exercises and equipment and would need education (i.e., visual guidebook) on establishing an MSA routine to limit overexertion. Some noted physical health issues as a barrier to MSA but felt they would be able to increase their activity levels with proper education on exercise modifications. Accessibility was another barrier mentioned. Most participants believed an MSA routine would be difficult to fit into daily routines due to limited time and gym accessibility. Others noted cost as a barrier and indicated a preference for joining an affordable gym.

#### Theme 3: Prior experience and familiarity with MSA guided current preferences for MSA interventions

Most participants associated MSA with lifting weights including both dumbbells and weight machines. However, when prompted with other MSA forms, such as Pilates and kickboxing, almost all participants responded with exercises they were familiar with beyond lifting weights. Physically active participants preferred an intervention that could be integrated into their current exercise routine. Participants without a current routine were open to trying new MSA or using equipment they were familiar with.

#### Theme 4: Desire for support and accountability

Overall, participants voiced their need for various forms of social support. Those who had participated in group-based PA spoke highly about the effect peers had on holding them accountable and motivating them to reach their goals. Others identified broad support systems (i.e., family, friends, etc.) as helpful for accountability purposes and supporting them in problem-solving challenges and barriers. Some participants mentioned their desire to connect with other survivors and relate to someone with a shared understanding of the cancer experience.

#### Theme 5: Need for flexible, convenient interventions with varied delivery, structure, and personalization

Convenient, tailored, and flexible interventions were a priority for participants. While mixed feedback was received on preferred delivery mode, participants were interested in both at-home and in-person workouts with recording features. Addressing accessibility in multiple ways, including physical, financial, technological, and time was regarded as a primary facilitator to engagement and MSA program sustainability. A motivation catalyst for participants was learning the rationale for specific exercises such as understanding their functional benefits. Exercise enjoyment was also strongly endorsed. Participants recommended prioritizing instruction via a multifaceted and tailored intervention, varying in difficulty and incorporating modifications to reduce injury risk and prevent overexertion with guidance on appropriate progression.

## Discussion

Overall**,** participants believed survivors should engage in MSA and it would be beneficial to their health. The majority indicated they were interested and physically able to participate in an MSA intervention but noted some potential barriers and ~50% of the sample met the MSA guidelines. Participants were interested in both in-person and technology-supported MSA intervention features and expressed the desire for MSA interventions to be user-friendly, tailored to survivors, flexible, personalized, and provide supportive accountability. Participants were most interested in engaging in MSA to improve their mental health, muscle tone, strength, energy, and balance, aiming to return to similar functioning as before their diagnosis. These data can help inform future MSA interventions for survivors.

Overall, our findings were similar to previous studies examining survivors’ general or MVPA intervention preferences [24, 25, 27, 33–35, 40–42]. Survivors were interested in MSA participation, which is consistent with preference studies in young adult and prostate cancer survivor populations [24, 25]. The observed preference for MSA interventions within 6 months to 1 year post-treatment is consistent with other studies on PA preferences [35]. However, 3–6 months was the most common timeframe in studies focused on general PA, suggesting survivors may feel they need more time post-treatment before beginning MSA interventions [35]. Still, over 70% were interested in MSA at every point along the cancer continuum. Additionally, our findings reflected variation on specific MSA intervention preferences based on demographic (age, gender, race, ethnicity, income) and disease characteristics, similar to findings from related, mixed methods studies [33]. Future research should identify the best timing for MSA interventions to maximize uptake and health outcomes and build upon findings of this study potentially on demographic factors and treatment types. Our findings demonstrate interest in technology-supported and highly tailored MSA interventions and are consistent with other studies demonstrating interest in technology-supported PA interventions, generally [41, 43]. Like other studies, participants were very interested in monitoring progress with wearables [33–35].

We observed variability among MSA intervention preferences consistent with previous studies demonstrating general PA preferences may vary by cancer type, gender, and age [35]. Participants in our study preferred to do MSA alone or in a hybrid format with some group and some individual sessions. In contrast, prior studies found individual exercise was the most preferred modality [35]. This may suggest group-based MSA programs may be viewed differently/more favorably than group-based aerobic sessions and is consistent with our qualitative findings demonstrating survivors believed exercise programs including social support from other survivors would be beneficial. Like other studies, we found variation in MSA location preference. However, ~50% were interested in receiving MSA instruction at a location other than home, which differs from other studies focused on general PA where most participants prefer a home-based program [35]. Similarly, about half of our sample preferred some MSA supervision, whereas most general exercise preferences studies found participants preferred unsupervised exercise [35]. These findings suggest survivors may desire more instruction or equipment than available at home, which was corroborated with our qualitative findings.

Regarding MSA equipment, results were variable but skewed towards resistance bands/free weights (51.8%) and free weights (65.3%). Participants were more likely to own free weights (70.9%), which may have influenced responses. Regardless, free weights and resistance bands are relatively inexpensive and may be accessible tools for both supervised and unsupervised MSA interventions. There was also interest in using commercially available smart equipment (Tonal, Peloton, Tempo) for MSA. Only one intervention to date has incorporated these applications with survivors [44]. As such, this is an emerging interest with a potential to offer increased safety monitoring and tailoring to enhance at-home MSA uptake and minimize burden associated with in-person MSA (i.e. travel time, supervision, parking, etc.).

Findings should be interpreted within the context of study limitations. Although a large sample size with varied cancer diagnoses was surveyed, participants were recruited post-treatment but within 5 years of diagnosis via medical records at a large academic medical institution. Additionally, while the proportion of Non-White and Hispanic survivors in our sample (38%) is consistent with the U.S. population (32%−36%), our sample was highly educated [45]. Thus, findings may not be representative of survivors from community settings or those from more socioeconomically disadvantaged backgrounds. Findings may also not represent potential heterogeneity among survivors of specific cancer types due to low enrollment, rarity, recruitment strategies, or prognosis. Further, the proportion of the sample meeting MSA and MVPA guidelines in our study (33.6%) was higher than in a population-based sample of cancer survivors (14%) [19]. Individuals who were more active may have been more likely to participate in this study since the focus was on interests and preferences of activity. We intentionally did not restrict participation based on activity level which resulted in variability in physical activity participation, but the sample, as a whole, was more active than population-based estimates. However, we used self-reported measures which have a higher potential risk of misclassification than objective measures, especially since the GLTEQ does not explicitly instruct participants to only list aerobic activities. While none of the specific intervention feature ratings were associated with meeting MSA or MVPA guidelines, findings should be interpreted within this context as the perspectives of those who are completely inactive may differ [19]. Our survey and interview did not include visuals or prototypes, which may impact participants’ question interpretation. Future studies should consider providing these resources to allow for more standardized survey and interview item interpretation. Finally, this study involved a cross-sectional assessment of interest and preferences for MSA interventions with a survey limited to what we believed, based on the literature, to be most important in an initial evaluation. As many factors may influence interests and preferences, future work should explore individual intervention preference dynamics to ensure intervention success and other intervention feature preferences that extend and expand our findings.

Study strengths include focusing on understanding the facilitators, barriers, needs, and preferences specific to MSA. Our sample size was relatively large with diversity in cancer type, age, gender, race and ethnicity. This study used both quantitative and qualitative techniques to capture survivors’ individual experiences and did not restrict by current MSA participation to explore preferences across activity levels. By understanding survivors’ MSA intervention preferences across various features, findings may increase future MSA intervention success in this population across settings and resources.

In conclusion, this study indicates cancer survivors of varying activity levels are interested in participating in MSA interventions using a wide range of potential components. Findings highlight the need to develop accessible, encouraging, and flexible interventions tailored specifically to survivors. The observed variability in survivors’ preferences for specific features indicates engaging survivors in designing, testing, and implementing MSA interventions is needed to enhance relevancy and efficacy. Future work is warranted to determine whether findings are consistent in cancers survivors who are more inactive and racially, ethnically and socioeconomically diverse and identify which MSA intervention features are most feasible, effective, engaging, and cost-effective for increasing and maintaining MSA and improving health outcomes among survivors.

## Supplementary Information

Below is the link to the electronic supplementary material.

ESM 1 (DOCX 14.4 KB)

ESM 2 (DOCX 47.9 KB)

## Data Availability

No datasets were generated or analysed during the current study.
