# Supplementary material for: Cancer survivors’ interests and preferences for muscle-strengthening activity interventions
Source: Support Care Cancer. 2026 Oct 9;34(11):1078. doi: 10.1007/s00520-026-11312-7 (PMC13652929; doi:10.1007/s00520-026-11312-7)
Supplement: Supplementary file 1 [file 520_2026_11312_MOESM1_ESM.docx]

**Supplemental Table 1. Interview Questions**

**Interview Questions:**

**Cancer and Physical Activity History**

1. Can you please tell me a little bit about your cancer diagnosis and the treatment you had?

2. What does your current physical activity routine look like? What did it looks like in the past?

3. What do you think is your biggest barrier in staying active with your routine?

4. What is your experience with fitness apps and fitness trackers? Have you used these devices? Would you be open to using one as part of a study? What would be barriers to you using these devices?

**Interview Questions:**

**Muscle-Strengthening Activities**

1. Do you currently do any activities specific to muscle-strengthening? If yes, what are these activities? If no, why not?

2. Have you ever had a regular muscle-strengthening exercise routine? If yes, what did you do? If not, why not?

3. What do you see as major barriers to muscle-strengthening exercise for yourself? For other cancer survivors?

4. What do you think could help facilitate participation in a muscle strengthening program for you and other cancer survivors?

5. If there was an option to enroll in a muscle-strengthening training intervention, what would your ideal intervention look like?

6. We are working on designing on distance-based muscle-strengthening intervention for cancer survivors. Do you think a program like this would be useful? Why or Why not?

7. What features of a distance-based muscle-strengthening program do you think would motivate you to participate?

8. Is there anything else you think the study team should consider in trying to design a muscle-strengthening intervention for cancer survivors?
