# Supplementary material for: Cancer survivors’ interests and preferences for muscle-strengthening activity interventions
Source: Support Care Cancer. 2026 Oct 9;34(11):1078. doi: 10.1007/s00520-026-11312-7 (PMC13652929; doi:10.1007/s00520-026-11312-7)
Supplement: Supplementary file 2 [file 520_2026_11312_MOESM2_ESM.docx]

**Supplementary Table 2. Participant Characteristics and Preferences for Features (n=553)**

|  | **Feature** | | | | | | | | |
| --- | --- | --- | --- | --- | --- | --- | --- | --- | --- |
| **Characteristic (Reference)** | **In person individual MSA training sessions**  **β (S.E.)** | **In-person group MSA training sessions**  **β (S.E.)** | **Individual video conference MSA training sessions**  **β (S.E.)** | **MSA activity data shared with doctor**  **β (S.E.)** | **Group video conference, live MSA training sessions**  **β (S.E.)** | **Website with MSA videos designed for cancer survivors**  **β (S.E.)** | **Smartphone app for MSA designed for cancer survivors**  **β (S.E.)** | **Smartphone app with MSA training videos designed for cancer survivors**  **β (S.E.)** | **Integration with social networking sites**  **β (S.E.)** |
| Gender (F, Ref= Male) | .12 (.14) | .16 (.15) | **.43 (.16)*** | -.17 (.17) | **.50 (.16)*** | .23 (.16) | .02 (.16) | -.03 (.16) | .15 (.18) |
| Race (White) | **-.32 (.14)*** | **-.38 (.15)*** | **-.33 (.16)*** | -.23 (.17) | -.32 (.16) | -.11 (.16) | .19 (.16) | .23 (.16) | -.22 (.18) |
| Ethnicity (Not Hispanic/Latin) | -.35 (.18) | **-.61(.18)**** | -.01 (.20) | -.05 (.08) | -.19 (.20) | -.11 (.20) | -.17 (.20) | -.25 (.20) | -.40 (.22) |
| Age | -.01 (.00) | .00 (.00) | **-.01 (.01)*** | -.10 (.16) | -.01 (.01) | -.00 (.01) | **-.03 (.01)**** | **-.03 (.00)**** | -.01 (.01) |
| BMI (Overweight/Obese (Ref= Normal) | -.09 (.35) | -.04 (.39) | .15 (.36) | .59 (.36) | .06 (.40) | -.08 (.34) | .23 (.38) | .05 (.37) | -.15 (.38) |
| Education | -.06 (.07) | -.015 (.07) | .01 (.07) | -.05 (.08) | -.04 (.07) | -.03 (.07) | -.04 (.07) | -.04 (.07) | **-.18 (.10)*** |
| Income $≤100k (Ref= >$100k, n=535) | -.02 (.09) | .17 (.09) | -.07 (.10) | .02 (.10) | .05 (.10) | .08 (.09) | -.01 (.10) | .01 (.10) | **.39 (.10)**** |
| Received Chemotherapy (Yes) | -.18 (.14) | -.18 (.15) | -.21 (.16) | -.16 (.27) | -.14 (.16) | .19 (.16) | .12 (.16) | .05 (.16) | -.13 (.17) |
| Received Radiation (Yes) | .25 (.13) | .27 (.14) | .06 (.15) | .31 (.20) | .27 (.15) | -.13 (.15) | .13 (.15) | .20 (.15) | .10 (.17) |
| Received Immunotherapy (Yes) | .22 (.23) | .21 (.24) | .05 (.26) | -.16 (.27) | .24 (.26) | -.05 (.25) | .07 (.26) | .11 (.26) | .45 (.28) |
| Received Surgical treatment (Yes) | **.41 (.20)*** | .32 (.20) | .12 (.22) | -.27 (.23) | .07 (.22) | -.13 (.22) | .11 (.22) | -.05 (.22) | -.19 (.24) |
| Health Status | -.01 (.08) | .01 (.09) | .02 (.10) | -.07 (.10) | -.04 (.09) | .13 (.09) | .06 (.09) | .01 (.09) | -.06 (.10) |
| Meet MVPA Guidelines | .24 (.14) | .21 (.14) | -.11 (.16) | .17 (.16) | .04 (.16) | .00 (.15) | -.02 (.15) | -.03 (.15) | .31 (.17) |
| Meet MSA Guidelines | .00 (.00) | -.00 (.00) | .00 (.00) | -.00 (.00) | -.00 (.00) | .00 (.00) | .00 (.00) | .00 (.00) | .00 (.00) |

|  | **Feature** | | | | | | |
| --- | --- | --- | --- | --- | --- | --- | --- |
| **Characteristic (Reference)** | **Activity Tracker**  **β (S.E.)** | **Access to “smart” MSA equipment**  **β (S.E.)** | **Exercise accountability buddy from personal life**  **β (S.E.)** | **Exercise accountability buddy from MSA activity program**  **β (S.E.)** | **Personalized text messages**  **β (S.E.)** | **Personalized emails**  **β (S.E.)** | **Integrations with electronic health record**  **β (S.E.)** |
| Gender (F, Ref= Male) | .20 (.16) | -.14 (.15) | .28 (.18) | .28 (.18) | **.42 (.19)*** | .**39 (.18)*** | -.25 (.17) |
| Race (White) | .12 (.16) | -.06 (.15) | **-.61 (.18)**** | **-.66 (.18)**** | -.26 (.19) | -.21 (.18) | -.09 (.17) |
| Ethnicity (Not Hispanic/Latin) | **-.43 (.19)*** | -.21 (.19) | **-.46 (.22)*** | **-.53 (.22)*** | -.16 (.23) | .05 (.23) | **-.55 (.21)**** |
| Age | **-.02 (.01)**** | **-.03 (.00)**** | **-.02 (.01)**** | -.01 (.01) | -.01 (.01) | -.002 (.01) | -.01 (.01) |
| BMI (Overweight/Obese (Ref= Normal) | -.23 (.38) | .22 (.36) | .83 (.43) | .30 (.43) | .69 (.41) | .80 (.41) | **.80 (.39)*** |
| Education | .04 (.07) | .04 (.07) | -.02 (.08) | -.04 (.08) | -.24 (.08) | **-.27 (.08)**** | -.00 (.10) |
| Income $≤100k (Ref= >$100k, n=535) | .04 (.10) | .02 (.10) | .16 (.11) | .20 (.11) | **.36 (.11)**** | **.35 (.11)**** | -.03 (.10) |
| Received Chemotherapy (Yes) | .03 (.15) | -.03 (.15) | **-.46 (.17)**** | **-.43 (.17)*** | -.06 (.18) | -.02 (.18) | .02 (.16) |
| Received Radiation (Yes) | .19 (.14) | **.29 (.14)*** | .25 (.16) | **.35 (.16)*** | .17 (.17) | .20 (.17) | .19 (.09) |
| Received Immunotherapy (Yes) | .10 (.25) | .04 (.24) | .25 (.28) | .47 (.28) | -.13 (.29) | -.10 (.29) | -.04 (.27) |
| Received Surgical treatment (Yes) | .13 (.21) | **.42 (.21)*** | -.14 (.24) | .18 (.24) | .16 (.25) | .23 (.25) | .00 (.00) |
| Health Status | .07 (.09) | -.03 (.09) | -.13 (.10) | -.16 (.10) | .01 (.10) | .08 (.10) | -.07 (.09) |
| Meet MVPA Guidelines | -.04 (.15) | -.02 (.14) | .19 (.17) | .14 (1.7) | -.01 (.18) | .08 (.17) | .24 (.16) |
| Meet MSA Guidelines | .00 (.00) | -.00 (.00) | .00 (.00) | .00 (.00) | .00 (.00) | .00 (.00) | -.00 (.00) |

***Note:*** Values in bold indicate factor is statistically significant. * indicates significant at *p<*0.05, and **** indicates significant at *p<*0.01.
